# Doping of PDMS-NQS Sensors to Modify Their Response and Sustainability: Ammonia Quantitation in Farm Atmospheres as a Case Study

**DOI:** 10.3390/polym17182466

**Published:** 2025-09-12

**Authors:** Belén Monforte-Gómez, Camila Soto, Pilar Campíns-Falcó

**Affiliations:** MINTOTA Research Group, Departament de Química Analítica, Facultat de Química, Universitat de València, 46100 Valencia, Spain; belen.monforte@uv.es (B.M.-G.); casoso@alumni.uv.es (C.S.)

**Keywords:** ammonia, composite, sensor, ionic liquid, deep eutectic solvent, choline chloride, NQS, farm atmosphere, UHPLC-QTOF

## Abstract

In this work, different passive solid composites of 1,2-naphthoquinone-4-sulfonic acid sodium salt (NQS) embedded in polydimethylsiloxane (PDMS) and tetraorthoethylsilicate (TEOS) doped with silica nanoparticles (SiO_2_NPs) were obtained. New composites including deep eutectic solvent (DES) and choline chloride (ChCl) were synthetized and compared here vs. ionic liquid (IL) which was previously proposed, from their passive response with time. Monitoring and controlling of ammonia levels inside poultry and rabbit farming facilities are essential for animal welfare, workers’ exposure assessment, and measurement of environmental emissions. Real poultry and rabbit farm atmosphere samples were analyzed at different sensor exposition times, obtaining results between two and eight ppmv of NH_3_ in all cases. The results were compared by air sampling with Tedlar bags and analysis by UHPLC-QTOF from a miniaturized SPE supported derivatization that was developed. As primary amine group NH_3_ was the major component in the farm atmosphere, the presence of methylamine was negligible. PDMS-based sensors with DES or ChCl add new potential for previously developed composites, improving the versatility for controlling ammonia by using new sustainable composites with different time responses.

## 1. Introduction

Ammonia (NH_3_) is one of the gases considered as a pollutant at certain emission levels, which can be harmful to humans, the environment and the industrial, agricultural, and livestock sectors [1]. Focusing on the livestock industry, NH_3_ levels are influenced by the decomposition of organic matter, particularly livestock manure and urine. Nitrogen is excreted in the form of NH_3_, uric acid, and urea, the latter being hydrolyzed into NH_3_ by various microbial enzymes [2]. Additionally, food waste and feathers in the poultry industry [3,4] contribute to NH_3_ levels. This negatively affects animal immunity and decreases the performance of livestock farms [5,6]. For these reasons, the control and monitoring of ammonia levels in farm atmospheres are increasingly emphasized, including this analyte in programs for the reduction and control of emissions of atmospheric pollutants [7,8]. The Council Directive 2007/43/EC [9] states that NH_3_ concentration should not exceed 20 ppmv over any eight-hour period in a poultry farm or 35 ppmv over any ten-minute period during the poultry production cycle. The established safety threshold in rabbit farms was of 10 ppmv [10].

To address this issue, our group, MINTOTA (Miniaturization and total analysis methods), has developed and patented different passive solid composites of 1,2-naphthoquinone-4-sulfonic acid sodium salt (NQS) embedded in polydimethylsiloxane (PDMS) and tetraorthoethylsilicate (TEOS) doped with silica nanoparticles (SiO_2_NPs) [11,12,13] and an ionic liquid (IL) [14]. Deep eutectic solvents (DESs) and named natural deep eutectic solvents (NADESs, mix of components that exist in nature) were studied here as new doping agents. DESs and NADESs have physicochemical properties very similar to those of ionic liquids; nevertheless, its preparation is much simpler as it uses readily available raw materials, presenting greater biodegradability. DESs and NADESs consist of mixtures formed by a hydrogen bond acceptor (HBA) compound, such as choline chloride (ChCl), and a hydrogen bond donor (HBD), such as formic acid, lactic acid, or glycerol, among others connected through hydrogen bonds or other non-covalent interactions [15,16,17,18,19,20]. These solvents, particularly those based on ChCl, exhibit comparable absorption capacities to ILs indicated in different references [21,22,23,24,25,26]. DESs are new green solvents that have opened a new field in sustainable processes. They have gained great attention as an ecofriendly, cost effective, and reusable alternative media to organic solvents an ILs, this is why the applications of DESs in diverse fields of science have grown in the last lustrum [27].

The NQS encapsulation is carried out in a PDMS polymer matrix modified with tetraethyl orthosilicate (TEOS) to impart hydrophilicity to the membrane [11,13,28,29]. The addition of silica nanoparticles (SiO_2_ NPs) to this mixture helps the sensor to gel, enhances the performance of the device as well as its sensitivity by improving the porosity and diffusion of the analyte into the polymer matrix [12,28,30]. The polymer matrix doped with different compounds including the IL 1-methyl-3-octylimidazolium hexafluorophosphate (OMIM PF6) was previously introduced in [14]. Here, a DES based on choline chloride and formic acid (ChCl-FA) and ChCl were synthetized by studying their response to ammonia. The hypothesis was to improve sensitivity and sustainability of the NQS membranes. General advantages of DESs over that of conventional solvents and ILs include low cost and toxicity, higher biodegradability, recyclability, wide stability, greenness, simple preparation, high diversity, and safety advantages [27]. ChCl with DESs and NADESs do not exhibit significant levels of cytotoxicity as indicated in several papers [31,32]. Their structural characteristics were studied using optical microscopy and scanning electron microscopy (SEM). These sensors were applied in poultry and rabbit farms for the determination of NH_3_ in their atmospheres. A confirmation study of the results obtained from proposed sensors were carried out from air sampling with Tedlar bags, a miniaturized SPE supported derivatization developed and ultra-high performance liquid chromatography coupled with quadrupole time-of-flight mass spectrometry (UHPLC-QTOF). A discussion about how doping the PDMS-NQS passive sensors provides versatility in sampling plans of ammonia measuring in several atmospheres is carried out.

## 2. Materials and Methods

### 2.1. Reagent Solutions and Materials

The Sylgard^®^ 184 silicone elastomer kit (consisting of a base and curing agent) used for the manufacture of the polydimethylsiloxane (PDMS) polymer was supplied by Dow Corning (Midland, MI, USA). The reagents used included tetraethyl orthosilicate (TEOS, ≥99.0%), silicon dioxide nanoparticles (SiO_2_NPs, 99.5%, particle size 5–15 nm), sodium 1,2-naphthoquinone-4-sulphonate (NQS, 99.7%), 1-methyl-3-octylimidazolium hexafluorophosphate (OMIM PF_6_, ≥95%), formic acid (FA, 98–100%), lactic acid (LA, ≥98%), glycerol (Gly, ≥99%), methylamine (MA, ≥98%), sodium salicylate (SL, ≥98%), 20 mM bicarbonate (sodium bicarbonate, ≥99%) buffer pH 12, dansyl chloride (Dns-Cl, ≥99%), and choline chloride (ChCl, ≥98%), all supplied by Sigma Aldrich (St. Louis, MO, USA). Sodium nitroprusside (NP, ≥95%) (Probus, Barcelona, Spain) and hydrochloric acid (HCl, 37%) (Scharlau, Barcelona, Spain). Sodium hypochlorite (NaOCl, 6–14%), acetone, and acetonitrile (ACN) (VWR Chemicals, grade LC-MS). The gaseous NH_3_ standard was prepared by mixing an ammonium chloride solution (NH_4_Cl, ≥99.5%) (Probus) and sodium hydroxide (NaOH, 98%) solution (Scharlau) in static dilution bottles (V = 2 L) (Supelco, Bellefonte, PA, USA). Air samples were collected using Tedlar^®^ bags (V = 2 L) (Supelco) and airtight bags of 2 L capacity from the Hardiron store (Shanghai, China).

### 2.2. Instruments

The morphology of the sensor was studied with a field emission scanning electron microscope (FESEM) SCIOS 2 FIB-SEMSCIOS 2 at an accelerating voltage of 5 kV, over metalized samples with a mixture of gold and palladium for 2 min.

To characterize both sides of the sensor, an optical microscope ECLIPSE E200LED MV Series (Nikon Corporation, Tokyo, Japan) equipped with brightfield illumination and 10× and 50× objectives was employed. Image acquisition was performed using the Nis Elements 4.20.02 software (Nikon Corporation, Tokyo, Japan).

Diffuse reflectance as absorption spectra of the colored sensors were recorded using a Varian Cary 60 UV–vis spectrophotometer equipped with a diffuse reflection probe from Harrick Scientific Products (Pleasantville, NY, USA). Spectra were recorded from 200 to 900 nm. For data collection and processing the CaryWinUV 5.1.3. software from Agilent Technologies was used. A wavelength of 590 nm was selected for quantitation.

Ultrasonic cleaner (Labbox, Mataró, Spain) was used to disperse compounds in the synthesis. Deep eutectic solvents were characterized by Fourier infrared (FTIR) spectroscopy using a Cary 630 FTIR (Agilent, Waldbronn, Germany).

An Agilent 6546 LC/QTOF (Agilent) equipped with an ESI ion source was used. The capillary and nozzle voltages were set at 3500 V and 500 V, respectively. Nitrogen, supplied by a Genius SQ 24 nitrogen generator (Peak Scientific Instruments Ltd., Ichinnan, UK) was utilized as the nebulized, drying, and sheath gas. The nebulizer gas pressure was maintained at 45 psi. For both the drying and sheath gas, the temperature was set at 300 °C with a flow rate of 10 L·min^−1^. Mass spectra were recorded in the range of 60 to 1500 *m*/*z* with an acquisition rate of 1.5 spectra·s^−1^ and an acquisition time of 666.7 ms·spectrum^−1^. Chromatographic separation was carried out using an Agilent 1290 Infinity II series system, equipped with an Eclipse Plus C18 RRHD analytical column (2.1 × 50 mm, 1.8 μm particle size) thermostatted at 35 °C. The mobile phase consisted of water/acetonitrile mixtures containing 0.1% formic acid for positive ionization. The gradient elution method was employed. The gradient started with 5% ACN, increased to 10% in the first minute, then to 90% by minute 5, maintaining this composition for 5 min. The total analysis was 10 min. The flow rate was set at 400 μL·min^−1^ and the injection volume was 5 μL.

### 2.3. Preparation of Deep Eutectic Solvents

DESs and NADESs containing choline chloride (ChCl) were synthesized using formic acid (FA) and glycerol (Gly) or lactic acid (LA) as hydrogen bond donors, respectively. These compounds were prepared using two different approaches starting from the procedures indicated in [15,17], respectively. In the first approach, ChCl:FA, ChCl:Gly, and ChCl:LA were synthesized and after several dilutions with water were carried out (Table 1). In the second approach, ChCl:FA:H_2_O and ChCl:LA:H_2_O were directly synthesized in water at different molar ratios as shown in Table 1. The mixtures of ChCl:FA, ChCl:Gly, ChCl:LA, ChCl:FA:H_2_O, and ChCl:LA:H_2_O were stirred and heated in a vial at 80 °C for 10 min to ensure homogeneity. The resulting DESs and NADESs were stored at 4 °C until their use.

For the characterization of DESs and NADESs, these solvents were dried in stove at 70 °C before FTIR measurement.

### 2.4. Synthesis of the Sensors

**Figure 1:**
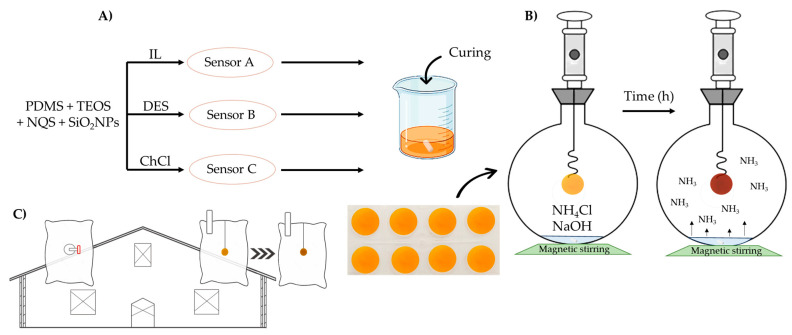
(**A**) Composite synthesis process, (**B**) ammonia gaseous standard prepared at the 2 L static dilution bottle. (**C**) Real farm sampling methods with Tedlar bags and hermetic bags with sensor inside them.

Synthesis of sensor A was carried out as in [14], with some modifications. The supported IL-based sensing membranes was performed by mixing the derivatizing reagent, NQS (0.3%), with the IL (7.7%). The mixture was then stirred at 700 r.p.m for 10 min. Then, the PDMS (35%) was added to the previous mixture and the resulting mixture was stirred for 10 min until homogenization (dispersion 1). On the other hand, a mixture of TEOS-SiO_2_NPs was prepared by mixing SiO_2_ NPs (1%) with TEOS (56%) by ultrasound for 10 s (dispersion 2). Finally, dispersion 1 and 2 were mixed and stirred vigorously to obtain a homogeneous mixture for around 4–5 h. Then, the curing agent (3.5%) was added (Table 2). Aliquots of 0.2 g of the mixture were taken and placed on a plastic mold with a circular mold 3 cm in diameter and 1 cm wide (Figure 1A), which were gelled at 40 °C for 24 h.

Synthesis of sensor B, the supported DES-based sensing membrane, was performed by mixing NQS (0.3%) with SiO_2_NPs (1%) and TEOS (56%), a dispersion was formed by ultrasounds for 10 s. Then, different DES and NADES compositions (7.7%) and PDMS (35%) were added to the mixture (Table 2). Finally, the dispersion was mixed and stirred vigorously to obtain a homogeneous mixture for 4–5 h. Then, the same process as sensor A was carried out.

Synthesis of sensor C, the supported ChCl based sensing membrane, was performed by mixing NQS (0.3%) with ChCl (0.5–5 M) (7.7%). The mixture was then stirred for 10 min. Then, the elastomer base PDMS (40–95%) was added to the previous mixture and the resulting mixture was stirred for 10 min to obtain a homogeneous dispersion (dispersion 1). On the other hand, a mixture of TEOS-SiO_2_NPs was prepared by mixing SiO_2_NPs (1%) with TEOS (0–60%) as Table 2 indicates, and the mixture was ultrasounded for 10 s (dispersion 2). Finally, the same process as sensor A was carried out.

The standard mixing ratio for PDMS was 10:1 elastomer and curing agent, respectively. This ratio provides the mechanical properties that are desirable and of optimum biocompatibility. Table 2 provides a concise summary of the synthesis procedures for each type of sensor.

### 2.5. Preparation of Ammonia Standards

The NH_3_ gas standards, with concentrations ranging from 1 to 50 ppmv, were generated in static dilution bottles with a volume of 2 L of air and evaluated at different sensor exposure times. Independent experimental setups were conducted for each combination of time and concentration. The ammonia atmosphere was created by combining 200 μL of NaOH (2 M) and 100 μL of an NH_4_Cl solution at a specific concentration, as illustrated in Figure 1B for the static dilution bottle. During sampling, the mixture was kept under continuous agitation.

After a defined exposure time, the sensor was removed from the static dilution bottle, and diffuse reflectance measurements between 550 and 700 nm and a wavelength of 590 nm for quantitation were performed. The absorbance difference between the standard and a blank reference, prepared with 200 μL of NaOH (2 M) and 100 μL of distilled water, was considered the sensor response. Gas standards were freshly prepared prior to each experiment to ensure their stability.

Additionally, the residue of the NaOH and NH_4_Cl solution was analyzed using a salicylate-nitroprusside colorimetric assay and UHPLC-QTOF (see next Section 2.6) to confirm that all the ammonium had been converted to ammonia within the static dilution bottle. The experimental procedure for UV-vis spectrophotometry consisted of mixing 200 μL of the residue of the standard solution with NaOH, 300 μL of H_2_O, 2 μL of sodium hypochlorite 2.3%, and 2 μL of the catalyst sodium nitroprusside [33]. Then, 2.5 μL of sodium salicylate was added and the corresponding indophenol blue was formed. The analytical determination of blue indophenol was performed by obtaining the absorption spectrum (λ = 690 nm) after 5 min.

The NH_3_ gas standards were also generated at a concentration ranging from 1 to 50 ppmv using Tedlar air bags with a capacity of 2 L. The NH_3_ atmosphere was prepared by combining 200 μL NaOH (2 M) and 100 μL NH_4_Cl solution at a specific concentration. The mixture was stirred to ensure adequate NH_3_ generation. After a fixed exposure time of 6 h, different volumes of air were withdrawn with a 60 mL syringe and derivatized as in the following section indicates. The residue of the NaOH and NH_4_Cl solution was analyzed using UHPLC-QTOF to confirm that all the ammonium had been converted to ammonia. In parallel, aqueous methylamine standards were also prepared and derivatized.

### 2.6. Derivatization of Ammonia and Methylamine in Gaseous Standards and Farm Air Samples

Tedlar bag air with a volume of 60 mL for each standard of sample was collected with a syringe and carefully transferred to a chromatography vial containing 1 mL of 0.1 M HCl. The final solution was then left for a period of 5 min and then neutralized with drops of 0.1 M NaOH.

**Figure 2:**
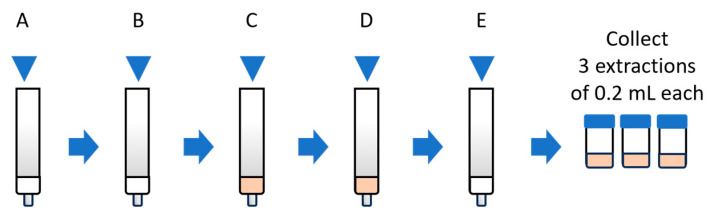
Schematic of the derivatization process for amine standards to UHPLC-QTOF: (**A**) preconditioning the cartridge, (**B**) standard loading, (**C**) derivatization with Dns-Cl, (**D**) washing of the cartridge, and (**E**) final elution of the derivatized compounds.

Solid phase extraction (SPE) supported derivatization using C18 cartridges 100 mg (Agilent, Waldbronn, Germany) was employed for analysis by UHPLC-QTOF. The SPE C18 cartridges were preconditioned with 0.5 mL methanol and 0.5 mL of 20 mM CO_3_^2−^/HCO_3_^−^ buffer solution of pH 12. Once conditioned, 0.5 mL of the standard or sample was taken from the vial and loaded onto the cartridge, followed by a drying step as indicated in Figure 2. Then, 0.25 mL of 3 mM Dns-Cl (previously prepared in a mixture of acetone and 20 mM CO_3_^2−^/HCO_3_^−^ buffer solution of pH 9, in volume concentration of 2:3% *v*/*v*) was passed through the cartridge. The derivatization was carried out at room temperature, in a light-protected environment, and the total reaction time was 10 min. After this time, the cartridge was washed with ultrapure water to remove impurities. Finally, the elution was carried out with three fractions of 0.2 mL acetonitrile, and each fraction was diluted five times with ultrapure water of mass spectrometry grade and measured (Figure 2), the analytical signal considered for quantitation was the addition of the three extraction signals. In parallel, aqueous methylamine standards were also prepared and treated according to the same derivatization protocol detailed above. Both analytes were analyzed by UHPLC-QTOF.

The percentage of the volatilization of different standard concentrations of NH_3_ in confined static dilution bottles and Tedlar bags was studied when mixing NH_4_Cl standard solutions of 498, 876, and 2190 ppm for 9.4, 18.8, and 47.8 ppmv NH_3_, respectively, and NaOH 2 M and volatilized in 2 L of air. These atmospheres were generated after 1 to 8 h. Afterwards, the remaining solutions were collected and analyzed by UV-vis spectrophotometry, as indicated in Section 2.5 and UHPLC-QTOF (see Section 2.2 also).

### 2.7. Real Farm Samples

Three bags of thirty air samples were collected from poultry (3) and rabbit (2) farms in several places in Spain to analyze ammonia concentrations using the two distinct sampling methodologies, as illustrated in Figure 1C. In the first protocol, Tedlar bags were employed to collect 2 L of farm air using a 500 mL syringe. The ammonia content in these samples was derivatized and subsequently analyzed using UHPLC-QTOF, following the protocol outlined previously.

In the second protocol, hermetically sealed bags containing a composite sensor were used. These bags were also filled with 2 L of farm air using a 500 mL syringe. The NH_3_ concentration was determined based on the analytical response of the sensor within the bag by UV-vis diffuse reflectance. Exposure times varied depending on the sensor type: 8 h for sensors A and B and 3 h for sensor C. The analytical signal was measured as absorbance via diffuse reflectance spectroscopy at 590 nm and the spectrum between 400 and 800 nm was also obtained for a qualitative proposal.

## 3. Results and Discussion

### 3.1. Characterization of Deep Eutectic Solvents

Different choline chloride-based deep eutectic solvents were synthetized for the development of sensor B, their composition is shown in Table 1. As indicated in Section 2.3, two approaches were employed, dilution of the trade mixtures with water and synthesis employing water. All of these DESs and NADESs were characterized by FTIR spectroscopy.

**Figure 3:**
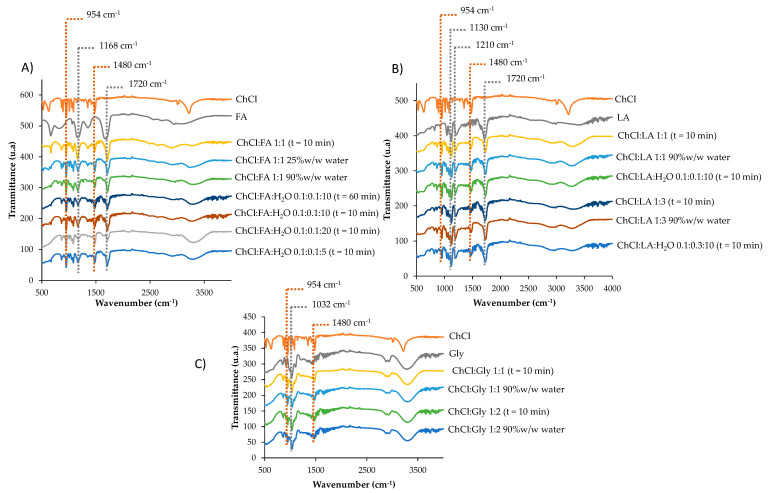
FTIR spectrum of different synthesis and composition of (**A**) ChCl:FA, (**B**) ChCl:LA, and (**C**) ChCl:Gly.

As shown in Figure 3A, the characteristics peaks of ChCl:FA appeared at 1720 (C=O), 1480 (CH_2_), 1168 (C-O),and 954 (C-N) cm^−1^ [16,34] with 10 min synthesis time. Peaks with similar transmittance appeared in this DES diluted up to 90% *w*/*w* in water and ChCl:FA:H_2_O 0.1:0.1:10 with a synthesis time up to 60 min. Moreover, similar results were observed with ChCl:FA:H_2_O 0.1:0.1:10 and ChCl:FA:H_2_O 0.1:0.1:5,; nevertheless, a reduction in transmittance peaks in ChCl:FA:H_2_O 0.1:0.1:20 was observed. In any case, all of the synthesized types of choline chloride and acid formic deep eutectic solvents showed the characteristic peaks of it.

Figure 3B shows the characteristics peaks of ChCl:LA 1:1, which appeared at 1720, 1480, 1210 (C-O), 1130 (C-O-H), and 954 cm^−1^ [18,34] with 10 min synthesis time. Peaks with similar transmittance appeared in the NADES diluted up to 90% *w*/*w* in water and ChCl:LA:H_2_O 0.1:0.1:10 with 10 min synthesis. Moreover, similar results were observed with ChCl:LA 1:3 and ChCl:LA:H_2_O 0.1:0.3:10. In all synthesized types of choline chloride and acid lactic natural deep eutectic solvents showed the characteristic peaks of it.

The characteristics peaks of ChCl:Gly 1:1 appeared at 1480, 1032 (C-O), and 954 cm^−1^ [18,34] with 10 min synthesis time. Similar results were observed in ChCl:Gly 1:2 and both NADESs diluted up to 90% *w*/*w* in water (Figure 3C).

### 3.2. Characterizing the Response of the Sensors A, B, and C

The influence of adding IL, DES-NADES, or ChCl into the polymeric matrix for sensor A, B, and C, respectively, was studied. Synthesis of sensor A was optimized in previous studies [14]; however, synthesis optimization of sensors B and C was carried out in this work.

For sensor B synthesis, the same percentages of PDMS:TEOS and SiO_2_NPs were used as in sensor A (see Table 2). Assays were carried out in static dilution bottles (V = 2 L air) for 6 h with a concentration of 18.8 and 28.3 ppmv NH_3_ for optimization of sensor B. Table 3 shows the influence of the order of addition of several compounds on the sensor gelation. As derived from Table 3, the synthesis B2 is the best option providing a homogenous membrane.

**Figure 4:**
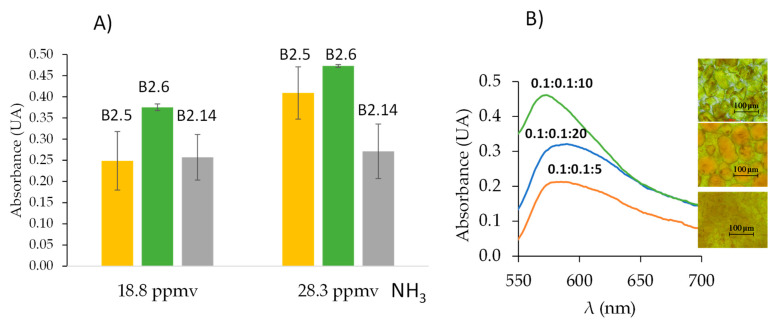
(**A**) Responses of B.2.5, B.2.6, and B.2.14 composites in static dilution bottles (V = 2 L) for 6 h with 18.8 and 28.3 ppmv NH_3_ (λ = 590 nm); *n* = 3 in all cases. (**B**) Spectra of ChCl:FA:H_2_O composites in static dilution bottles (V = 2 L) for 6 h with 18.8 ppmv NH_3_ (λ = 590 nm); inserts correspond to bright-field microscopy photographs of 10× magnification obtained for sensing membrane before ammonia exposition.

Different types and compositions of deep eutectic solvents such as ChCl:FA, ChCl:LA, and ChCl:Gly as Table 4 shows, were tested with the objective to obtain comparable responses with those provided by sensor A. It was demonstrated that DESs and NADESs without high water content did not work because the sensor did not gel. Water can cause variations in the interactions of DESs within the sensor, allowing its gelation in the presence of a high percentage of water in the deep eutectic solvent [35]. Synthesis B2.5, B2.6, and B2.14 provided sensors with response to ammonia, seen in Table 4 and Figure 4A. However, synthesis B2.6 provided better precision (see Figure 4A). The deep eutectic solvent selected was ChCl:FA:H_2_O 0.1:0.1:10 with a synthesis time of 10 min. Other molar ratios were tested: 0.1:0.1:20 and 0.1:0.1:5 but the ammonia responses were lower as Figure 4B shows, the morphology of the membranes were different (see inserts of Figure 4B).

For the synthesis of sensor C, an additional order of reagents was studied from assays of the sensor in static dilution bottles (V = 2 L air) for one or two h with a concentration of 18.8 ppmv NH_3_. These syntheses are described in Table 5, selecting synthesis C1 for further assays because the membrane obtained was homogenous.

Additionally, PDMS:TEOS percentages were studied, as Table 6 indicates, 50:50% was selected for the following assays. In addition, the concentration of ChCl was studied up to 5 M, and 1 M was selected for this study. The selected synthesis was C1.4, which provided the highest absorbance values and good precision, as can be seen in Table 6.

**Figure 5:**
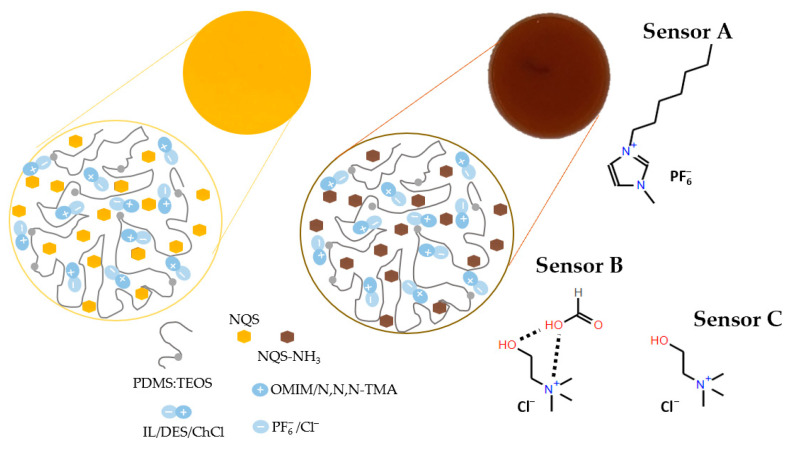
Schematic diagram of sensing membrane composition and its response: (sensor A) OMMIM PF6, (sensor B) ChCl:FA:H_2_O 0.1:0.1:10, (sensor C) ChCl.

Figure 5 shows a schematic diagram of sensing membrane compositions and their responses for (sensor A) OMMIM PF6, (sensor B) ChCl:FA:H2O 0.1:0.1:10, (sensor C) ChCl. For sensor A, which contained an ionic liquid with an imidazolium cation and a PF_6_^−^ anion, simultaneous hydrogen bonds between PF_6_, and silica matrix together with imidazolium groups π-π stacking interactions can occur [14]. For sensor B and C, the interactions are probably due to the Ch^+^ cation and Cl^−^ anion, through a physical interaction between silanol group and Cl^−^, which in turn disrupts the electrostatic interaction between Ch^+^ and Cl^−^ [36].

**Figure 6:**
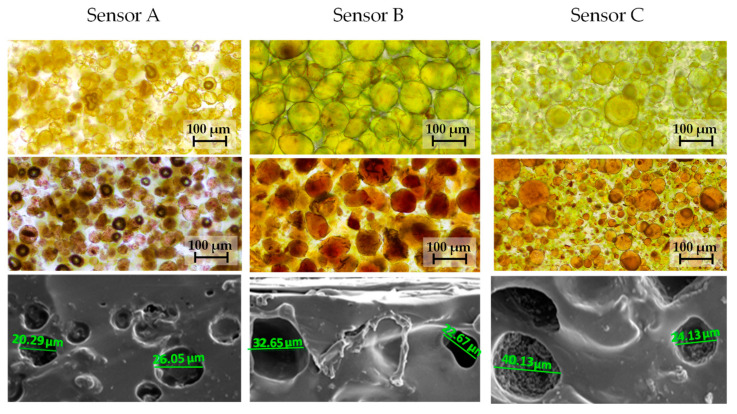
Bright-field microscopy photographs of 10× magnification obtained for sensing membrane before and after ammonia exposition for sensor A, sensor B, and sensor C. SEM micrograph of the transversal cut of the polymetric membrane of sensor A, sensor B, and sensor C.

The morphology presented some differences between the three sensors, as can be derived from Figure 6, which was obtained from bright-field microscopy photographs and SEM micrographs. The porosities are more similar for sensors B and C.

**Figure 7:**
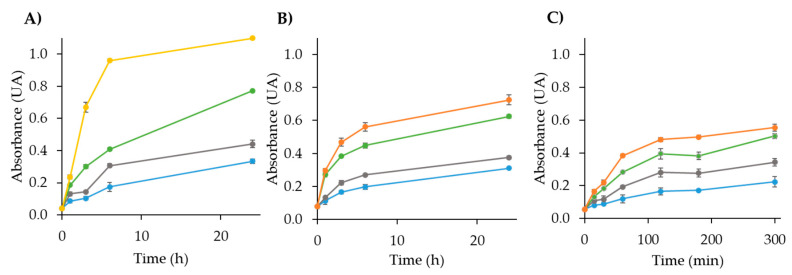
Variation in the sensing response of NH_3_ at several levels of concentration (ppmv) as a function of the exposure time with (**A**) sensor A, (**B**) sensor B, and (**C**) sensor C in different ammonia concentrations, 4.7 ppmv (blue), 9.4 ppmv (gray), 18.8 ppmv (green), 28.3 ppmv (orange), and 47.2 ppmv (yellow).

The kinetic reactions between NH_3_ and the composites A, B, and C were studied. Kinetic data were collected by measuring the absorbance of the sensors at various exposure times and ammonia concentrations at room temperature, with a two L air sampling volume. The results are presented in Figure 7. The effect of ammonia concentration on the reaction rate for sensors A and B was evaluated over 24 h at a concentration of 4.72 ppmv NH_3_, which followed first-order kinetics. The slopes of the linear regressions of Ln(Abs) versus time (hours) (*n* = 6) were used to determine the initial reaction rates, calculated as 0.055 ± 0.012 h^−1^ for sensor A and 0.040 ± 0.011 h^−1^ for sensor B. For sensor C, the kinetics were studied over five h, yielding a slope of 0.0035 ± 0.0006 h^−1^ for the linear regression of Ln(Abs) versus time (hours) (*n* = 6). Langmuir adsorption isotherms were examined to characterize the interaction between the sensors and ammonia. A plot of 1/A (where A represents absorbance, proportional to the amount of ammonia adsorbed by the sensor) versus 1/ammonia concentration (ppmv) was generated for sensors A and B at 3, 8, and 24 h, and for sensor C at 1, 3, and 5 h. The slopes of these plots are summarized in Table 7, with R^2^ values exceeding 0.95, demonstrating the reliability of the Langmuir model in describing the adsorption behavior.

In the case of sensor A, the uptake of ammonia exhibited a progressive increase between three and eight h of exposure. In contrast, for sensor B, the caption levels remained similar between three and eight h. Notably, the adsorption observed in sensor B after three h was comparable to that of sensor A after eight h, indicating that sensor B showed enhanced ammonia uptake efficiency over a reduced exposure time. For sensor C, a similar capture behavior was observed between three and five h, suggesting that ammonia uptake reached equilibrium by three h, as no significant variation in the slope was detected at five h. Additionally, the slope values for 24 h assays were similar for both sensors A and B, reinforcing the idea of saturation at longer exposure times.

As shown in Figure 7, sensor C achieved equilibrium faster than sensor B, while sensor B reached saturation faster than sensor A. These findings indicate that sensors B and C demonstrated more efficient ammonia absorption in less time compared to sensor A, highlighting their suitability for applications requiring rapid response times.

### 3.3. Stablishing Analytical Parameters from the Response of Sensor A, B and C

The solutions used to generate the ammonia standards were analyzed according to Section 2.5 for obtaining the ammonia volatility percentage. The volatilization percentages of ammonium in ammonia under the test conditions studied were between 96 and 99% (*n* = 20, 98 ± 2). There were no significant differences between the use of the static dilution bottle and the Tedlar bag, so there is the ability to choose any of the two test vessels as required. The values were similar at different test times between one and eight h, thus ensuring volatilization of most of the ammonium present in ammonia to the confined atmosphere. Furthermore, the percentage of volatilization was similar regardless of the ammonia concentration used for the test. These results agreed with previous studies [14].

Analytical parameters such as sensitivities, limit of quantifications (LOQ), and limit of detections (LOD) at different assay times were obtained. Suitable regression coefficients in all studied cases and relative standard deviation, RSD < 8%, as shown in Table 8, were achieved.

For sensors A and B, the ionic liquid and the deep eutectic solvent resulted in comparable responses across different exposure times, achieving similar sensitivity and detection limits. This suggests that the deep eutectic solvent can replace the ionic liquid, leading to a more environmentally friendly sensor with lower cost and improved biodegradability, while maintaining equivalent performance under the same conditions. In the case of sensor C, similar sensitivities and better detection limits were achieved within shorter exposure times. As demonstrated previously, sensor C exhibited a faster NH_3_ uptake capacity compared to sensors A and B. These data indicated the capacity of the doping agents to determine the time-response and the amount of NQS and ammonia retained in the membranes, this versatility can be useful in sampling plan strategies.

### 3.4. Stability of the Three Synthetized Composites

**Figure 8:**
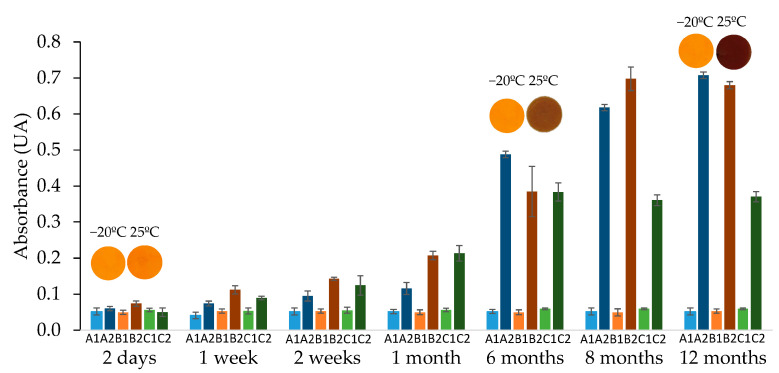
Analytical signal for blank studied composites for 12 months stored at −20 °C (marked as 1) and at 25 °C (marked as 2) for sensors A, B, and C. For more explanation see text.

At the lab, some composites were stored in a freezer at −20 °C, while others were kept at room temperature (25 °C) and exposed to atmospheric conditions. Blank absorption measurements were performed using diffuse reflectance for sensors A, B, and C under both storage conditions over a period of up to 12 months, as illustrated in Figure 8.

The stability of sensors stored in the freezer was demonstrated, at least, for 12 months, maintaining the analytical signal characteristic of the blank of each sensor during all this time stored at low temperatures. However, for composites exposed to the atmosphere at room temperature, the degradation of the NQS reagent started after one week of exposure.

The preservation of the sensors to maintain their stability for sampling in farms did not present any problems, as they remained stable for at least three months at 4 °C; the absorbance variation for non-exposed sensors (*n* = 20) was 0.06 ± 0.01 UA. All the sampled farms are equipped with a refrigerator capable of storing them until their use.

### 3.5. Application of Sensors in Real Poultry Farms

Measurements of air in two poultry and two rabbit farms were carried out by sampling, with a 500 mL syringe, two L of air inside farm ship in hermetically sealed bags, in which the sensor A, B, or C was inside, and Tedlar bags for a UHPLC-QTOF method developed here (see Section 2.5 and Section 2.6).

The linear working ranges for ammonia and methylamine obtained by UHPLC-QTOF were as follows: 0.05 (LOQ) − 50 ppmv and 0.05 (LOQ) − 10 ppmv and the calibration graphs: (−1.2 ± 0.1) + (56 ± 2) ppmv; R^2^ = 0.998 (*n* = 7) and (−0.3 ± 0.2) + (14.6 ± 0.7) ppmv; R^2^ = 0.998 (*n* = 7), respectively. The precision achieved was lower than 7% in all cases.

Sensors A and B were maintained for eight h and sensor C for three h inside bags at room temperature and after, diffuse reflectance as absorbances were measured. Some blanks of each sensor were assayed sampling atmospheric air from outside the farm building and any modification of the signal sensor was obtained.

The NH_3_ concentrations measured using UHPLC-QTOF and the sensors evaluated in this study showed comparable results across the 30 atmospheres analyzed as can be seen in Table 9. Layer, broiler, and rabbit farms with natural ventilation or cross-flow mechanical ventilation were sampled in winter, spring, or summer, as can be seen in Table 9. In all cases for each farm, the normal manure management was carried out. Some differences can be due to the possible lack of homogeneity of the farm atmosphere. Additionally, methylamine was studied by UHPLC-QTOF, and the presence was detected only in two farm atmosphere samples at concentrations close to the limit of detection, indicating NH_3_ was the predominant component in the farms (Table 9). These results are in accordance with [37].

The results obtained for samples S7, S8, and S9, which used sensors kept in farm at four °C during 21, 27, and 41 days before sampling corroborated their stability as indicated in the previous section, similar results for ammonia were achieved. NQS provided selectivity in reference to other family compounds because this reagent is a derivatization agent for primary and secondary amino groups. Ammonia in presence of hydrogen sulfide was tested in [14,38], and no effect was observed. This statement was also supported by the results obtained from the confirmatory study from air sampling in Tedlar bags and analysis by UHPLC-QTOF because comparable results were obtained (see Table 9).

## 4. Conclusions

In this work, a new synthetic strategy was investigated, in order to prepare colorimetric passive solid sensors that incorporate DESs and ChCl, as a replacement for ILs, which was studied in a previous work [14] and also in this study, to compare the performance of these composites under the same conditions. Morphological changes that these compounds provide to sensors which achieved improved or equalized sensitivity were successfully verified.

Results of adsorption can suggest that ammonia was adsorbed on homogeneous adsorption sensor sites, based on the Langmuir isotherm. Furthermore, calibration of passive sensors was carried out in function of sampling time and ammonia concentration which successfully allowed the establishment of analytical parameters such as sensitivity, limit of quantification, and limit of detection for sensors A (IL), B (DES), and C (ChCl). Stability of these sensing devices in time at different ways of storage were tested at a lab, stablishing that all three composites were conserved in perfect conditions at −20 °C stored conditions for at least one year. The stability of the sensors in the farms for at least three months at 4 °C was achieved.

These composites were based in PDMS as a polymer since it presents optimal properties for sensors purpose such as optical transparency, low manufacturing cost and toxicity, easy fabrication, flexibility, stretchability, and gas permeability; therefore, when a NQS-reagent is embedded inside, this PDMS-TEOS polymeric matrix can be considered a good strategy to develop different sensing devices with colorimetric response when these are exposed to ammonia concentrations, since this analyte diffuses inward thanks to its permeability. In addition, doping these sensing devices with ILs, DESs and ChCl increased the sensitivity and reduced limits of quantification and detection due to increased porosity that is produced in the composite combined with the ammonia absorption properties of these compounds. This versatility permits the use of several compositions in this function of the needs of the atmosphere that need to be controlled.

The study of ammonia in the atmosphere of poultry and rabbit farms as a use case was proposed. Layer, broiler, and rabbit farms with natural ventilation or cross-flow mechanical ventilation were sampled in winter, spring, or summer in order to quantify ammonia. Results were validated by UHPLC-QTOF, sampling farm air in Tedlar bags, obtaining similar values of ammonia concentration in farms with sensors doped with ionic liquid, deep eutectic solvents and choline chloride inside hermetically sealed bags with two L farm air, tested for eight (sensor A and B) or three h (sensor C), respectively. The presence of methylamine was also studied and it was concluded that the main component in these farm atmospheres was NH_3_. Results ranged from two to eight ppmv of NH_3_ as the legal limit allows 20 ppmv of ammonia in the air for poultry farms and 10 ppmv inside rabbit farming facilities. The employment of sensor B, with deep eutectic solvent ChCl:FA:H_2_O 0.1:0.1:10, and sensor C, with choline chloride, could be a new potential green and cost-effective alternative to in situ analysis and monitorization of ammonia levels in farms. DESs are new green solvents that have opened a new field in sustainable processes.

## Figures and Tables

**Table 1:** Deep eutectic solvents composition.

DES *	%H_2_O	DES **	mol/mol
ChCl:FA (1:1)	0	ChCl:FA:H_2_O	0.1:0.1:20
	25		0.1:0.1:10
	50		0.1:0.1:5
	75		
	90		
NADES *	**%H_2_O**	NADES **	**mol/mol**
ChCl:Gly (1:1)	0	ChCl:LA:H_2_O	0.1:0.1:10
	90		
ChCl:Gly (1:2)	0		
	90		
ChCl:LA (1:1)	0		0.1:0.3:10
	90		
ChCl:LA (1:3)	0		
	90		

* Synthetized and diluted in water later; ** synthetized in water.

**Table 2:** Mass composition of the different sensor types chosen. For more information see text.

	Sensor	(*w*/*w*)%
**NQS**	A–B–C	0.3
**IL/DES (ChCl:FA:H_2_O) (0.1:0.1:10)/ChCl (1M)**	A–B–C	7.7
**PDMS**	A–B	35
	C	45.5
**Curing**	A–B	3.5
	C	4.5
**TEOS**	A–B	56
	C	45.5
**SiO_2_NPs**	A–B–C	1

**Table 3:** Influence of order of addition of compounds in the synthesis of the sensor B.

Synthesis	Step 1	Step 2	Step 3	Step 4	Synthesis Gelling
B.1	NQS + DES/NADES	PDMS (40%)	SiO_2_NPs + TEOS (60%)	Curing agent	NQS agglomerated in beaker
B.2	NQS + SiO_2_NPs + TEOS (60%)	DES/NADES	PDMS (40%)	Curing agent	Homogenous
B.3	NQS + PDMS (40%)	-	SiO_2_NPs + TEOS (60%) + DES/NADES	Curing agent	NQS agglomerated in beaker
B.4	NQS + PDMS (40%)	DES/NADES	SiO_2_NPs + TEOS (60%)	Curing agent	NQS agglomerated in beaker

**Table 4:** Optimization of DES (ChCl:FA) and NADES (ChCl:LA and ChCl:Gly) composition for sensor B.

Synthesis	DES Type	NQS Polimerized	Gelled	Response
B.2.1	ChCl:FA (1:1)	No	No	
B.2.2	ChCl:FA (1:1) 25% *w*/*w*	No	No	
B.2.3	ChCl:FA (1:1) 50% *w*/*w*	No	No	
B.2.4	ChCl:FA (1:1) 75% *w*/*w*	No	No, higher viscosity	
B.2.5	ChCl:FA (1:1) 90% *w*/*w*	No	Yes	Yes
B.2.6	ChCl:FA:H_2_O (0.1:0.1:10)	No	Yes	Yes
B.2.7	ChCl:LA (1:1)	No	No	
B.2.8	ChCl:LA (1:1) 90% *w*/*w*	No	Yes	No
B.2.9	ChCl:LA:H_2_O (0.1:0.1:10)	No	Yes	No
B.2.10	ChCl:LA (1:3)	No	No	
B.2.11	ChCl:LA (1:3) 90% *w*/*w*	No	Yes	No
B.2.12	ChCl:LA:H_2_O (0.1:0.3:10)	Yes	Yes	No
B.2.13	ChCl:Gly (1:1)	No	No	
B.2.14	ChCl:Gly (1:1) 90% *w*/*w*	No	Yes	Yes
B.2.15	ChCl:Gly (1:2)	No	No	
B.2.16	ChCl:Gly (1:2) 90% *w*/*w*	Yes	Yes	

**Table 5:** Optimization of the order of additional compounds in the synthesis of sensor C.

Synthesis	Step 1	Step 2	Step 3	Step 4	Synthesis Gelling
C.1	NQS + ChCl 1M	PDMS (40%)	SiO_2_NPs + TEOS (60%)	Curing agent	Homogeneous
C.2	NQS + SiO_2_NPs + TEOS (60%)	PDMS (40%)	ChCl 1 M	Curing agent	NQS agglomerated in beaker
C.3	NQS + PDMS (40%)	-	SiO_2_NPs + TEOS (60%) + ChCl (1 M)	Curing agent	SiO_2_NPs agglomerated with ChCl
C.4	NQS + PDMS (40%)	ChCl (1M)	SiO_2_NPs + TEOS (60%)	Curing agent	NQS agglomerated

**Table 6:** Optimization of percentage PDMS/TEOS and ChCl molarity in the synthesis of sensor C; * (*n* = 6).

Synthesis	PDMS:TEOS (%)/ChCl (M)	Polimerized NQS	Gelled	Homogenous	Absorbance (*n* = 3)
C.1.1	100:0/1	Yes	No	-	-
C.1.2	80:20/1	No	No	-	-
C.1.3	60:40/1	No	Yes	Yes	0.278 ± 0.014
C.1.4	50:50/1	No	Yes	Yes	0.410 ± 0.010 *
C.1.5	40:60/1	No	Yes	Yes	0.39 ± 0.06
C.1.4.1	50:50/0.5	No	Yes	No	0.34 ± 0.03
C.1.4.2	50:50/2	No	Yes	Yes	0.297 ± 0.006
C.1.4.3	50.50/5	No	No	-	-

**Table 7:** Corresponding slopes to 1/A vs. 1/ppmv of ammonia for different composite types at times studied. Slope (ppmv).

Time Assay (h)	Sensor A		Sensor B		Sensor C	
	Slope	R^2^	Slope	R^2^	Slope	R^2^
1	-	-	-	-	32.748	0.99
3	43.093	0.95	23.324	0.96	11.516	0.96
5	-	-	-	-	10.726	0.96
8	23.308	0.98	22.141	0.99	-	-
24	9.1684	0.99	7.2148	0.95	-	-

**Table 8:** Some figures of merit for the different sensors (*n* = 6).

Sensor	Time Assay (h)	Linear Interval, LOQ–Higer C (ppmv)	Linearity (y = bx ± a)			LOD (ppmv)
			b ± Sb	a ± Sa	R^2^	
A	3	6–47	0.0132 ± 0.0003	0.040 ± 0.009	0.99	2.0
	8	3–47	0.020 ± 0.002	0.09 ± 0.06	0.99	1.0
	24	1.5–4.7	0.051 ± 0.003	0.090 ± 0.010	0.99	0.5
B	3	4.5–28	0.0112 ± 0.0008	0.12 ± 0.02	0.98	1.5
	8	3–28	0.0173 ± 0.0009	0.093 ± 0.012	0.99	1.00
	24	2–4.7	0.041 ± 0.004	0.115 ± 0.010	0.97	0.6
C	1	4.5–28	0.0121 ± 0.0006	0.052 ± 0.009	0.99	1.50
	3	2.5–28	0.0146 ± 0.0004	0.090 ± 0.004	0.99	0.9
	5	2.5–19	0.0217 ± 0.0011	0.109 ± 0.009	0.99	0.9

**Table 9:** Real farm air samples measured by UHPLC-QTOF and by sensor A, sensor B, and sensor C; *n* = 3, N.D. not detected; Layer farm (lf), broiler farm (bf), rabbit farm (rf), winter (w), spring (sp), summer (su), natural ventilation (nv), cross-flow mechanical ventilation (cf).

Sample	[NH_3_] ppmv				[CH_3_NH_2_] ppmv			
	UHPLC-QTOF	Sensor A	Sensor B	Sensor C	UHPLC-QTOF	Sensor A	Sensor B	Sensor C
**S1** **lf-** **w** **-nv**	3.3 ± 0.13	3.6 ± 0.2	3.3 ± 0.2	-	N.D.	N.D.	N.D.	N.D.
**S2** **lf-** **w** **-nv**	2.7 ± 0.2	3.0 ± 0.3	3.0 ± 0.3	-	~0.04	N.D.	N.D.	N.D.
**S3** **bf-su-cf**	3.0 ± 0.4	3.0 ± 0.3	3.0 ± 0.3	2.8 ± 0.2	~0.01	N.D.	N.D.	N.D.
**S4** **bf-su-cf**	2.8 ± 0.5	<LOQ	<LOQ	<LOQ	N.D.	N.D.	N.D.	N.D.
**S5** **bf-su-cf**	3.0 ± 0.1	<LOQ	<LOQ	<LOQ	N.D.	N.D.	N.D.	N.D.
**S6** **bf-su-cf**	3.1 ± 0.2	-	<LOQ	<LOQ	N.D.	N.D.	N.D.	N.D.
* S7 **bf-su-cf**	-	5.0 ± 0.2	5.9 ± 0.3	5.9 ± 0.3	-	N.D.	N.D.	N.D.
** S8 **bf-su-cf**	-	5.5 ± 0.3	5.9 ± 0.1	6.5 ± 0.4	-	N.D.	N.D.	N.D.
*** S9 **bf-su-cf**	-	4.9 ± 0.4	5.4 ± 0.3	5.9 ± 0.2	-	N.D.	N.D.	N.D.
**S10** **rf-sp-cf**	6.6 ± 0.8	4.3 ± 0.2	5.1 ± 0.2	-	N.D.	N.D.	N.D.	N.D.
**S11** **rf-sp-cf**	8.7 ± 0.1	6.0 ± 0.4	5.8 ± 0.1	5.3 ± 0.2	N.D.	N.D.	N.D.	N.D.
**S12** **rf-sp-cf**	7.7 ± 0.2	6.0 ± 0.3	4.5 ± 0.2	-	N.D.	N.D.	N.D.	N.D.
**S13** **rf-sp-cf**	7.72 ± 0.12	4.8 ± 0.13	4.5 ± 0.3	-	N.D.	N.D.	N.D.	N.D.
**S14** **rf-sp-cf**	7.33 ± 0.08	-	5.6 ± 0.3	-	N.D.	N.D.	N.D.	N.D.
**S15** **rf-sp-cf**	4.81 ± 0.09	3.6 ± 0.2	4.8 ± 0.3	5.4 ± 0.2	N.D.	N.D.	N.D.	N.D.
**S16** **rf-sp-cf**	3.2 ± 0.6	3.2 ± 0.2	2.8 ± 0.2	3.0 ± 0.2	N.D.	N.D.	N.D.	N.D.
**S17** **rf-sp-cf**	4.65 ± 0.11	3.60 ± 0.16	4.4 ± 0.2	3.8 ± 0.2	N.D.	N.D.	N.D.	N.D.
**S18** **rf-sp-cf**	4.9 ± 0.7	3.6 ± 0.2	3.9 ± 0.2	2.9 ± 0.2	N.D.	N.D.	N.D.	N.D.
**S19** **rf-sp-cf**	5.28 ± 0.04	4.2 ± 0.2	5.0 ± 0.2	5.2 ± 0.3	N.D.	N.D.	N.D.	N.D.
**S20** **rf-sp-cf**	4.9 ± 0.1	4.2 ± 0.3	4.3 ± 0.3	-	N.D.	N.D.	N.D.	N.D.
**S21** **rf-sp-cf**	7.0 ± 0.3	6.60 ± 0.16	-	6.4 ± 0.4	N.D.	N.D.	N.D.	N.D.
**S22** **rf-sp-cf**	5.03 ± 0.09	5.7 ± 0.3	4.7 ± 0.2	5.6 ± 0.4	N.D.	N.D.	N.D.	N.D.
**S23** **rf-sp-cf**	6.0 ± 0.4	6.5 ± 0.2	-	6.9 ± 0.4	N.D.	N.D.	N.D.	N.D.
**S24** **rf-sp-cf**	5.4 ± 0.5	5.6 ± 0.3	6.1 ± 0.4	6.2 ± 0.3	N.D.	N.D.	N.D.	N.D.
**S25** **rf-sp-cf**	4.5 ± 0.7	4.6 ± 0.2	4.6 ± 0.2	6.0 ± 0.3	N.D.	N.D.	N.D.	N.D.
**S26** **rf-sp-cf**	5.6 ± 0.1	5.8 ± 0.2	7.1 ± 0.4	8.2 ± 0.4	N.D.	N.D.	N.D.	N.D.
**S27** **rf-sp-cf**	5.3 ± 0.3	5.18 ± 0.15	3.4 ± 0.2	4.6 ± 0.3	N.D.	N.D.	N.D.	N.D.
**S28** **rf-sp-cf**	6.29 ± 0.08	6.4 ± 0.3	6.4 ± 0.3	5.87 ± 0.12	N.D.	N.D.	N.D.	N.D.
**S29** **bf-w-cf**	5.4 ± 0.3	6.43 ± 0.12	6.0 ± 0.5	5.41 ± 0.13	N.D.	N.D.	N.D.	N.D.
**S30** **bf-w-cf**	5.24 ± 0.08	5.7 ± 0.3	6.8 ± 0.4	5.8 ± 0.2	N.D.	N.D.	N.D.	N.D.

Sensors kept in farm at 4 °C for 21 days *, 27 days ** and 41 days *** before sampling.

## Data Availability

Data contained in the paper.
